# Tissue specific promoters improve specificity of AAV9 mediated transgene expression following intra-vascular gene delivery in neonatal mice

**DOI:** 10.1186/1479-0556-6-13

**Published:** 2008-09-23

**Authors:** Christina A Pacak, Yoshihisa Sakai, Bijoy D Thattaliyath, Cathryn S Mah, Barry J Byrne

**Affiliations:** 1 Powell Gene Therapy Center, College of Medicine, University of Florida, 1600 SW Archer Road, Gainesville, FL 32610-0266, USA

## Abstract

The AAV9 capsid displays a high natural affinity for the heart following a single intravenous (IV) administration in both newborn and adult mice. It also results in substantial albeit relatively lower expression levels in many other tissues. To increase the overall safety of this gene delivery method we sought to identify which one of a group of promoters is able to confer the highest level of cardiac specific expression and concurrently, which is able to provide a broad biodistribution of expression across both cardiac and skeletal muscle. The *in vivo *behavior of five different promoters was compared: CMV, desmin (Des), alpha-myosin heavy chain (α-MHC), myosin light chain 2 (MLC-2) and cardiac troponin C (cTnC). Following IV administration to newborn mice, *LacZ *expression was measured by enzyme activity assays. Results showed that rAAV2/9-mediated gene delivery using the α-MHC promoter is effective for focal transgene expression in the heart and the Des promoter is highly suitable for achieving gene expression in cardiac and skeletal muscle following systemic vector administration. Importantly, these promoters provide an added layer of control over transgene activity following systemic gene delivery.

## Findings

When developing gene therapy, it is important to minimize adverse responses to protein expression in unnecessary sites by restricting transgene expression to areas where it is most desirable. This confinement can be tissue restricted expression such as in the heart, or limited expression to a combination of tissues such as those affected in the muscular dystrophies.

One way to control the site of transgene expression is through choice of physical delivery route [1]. Direct injections into a specific tissue help to concentrate transduction to that exact location. However, it is often difficult to achieve a broad and even biodistribution of expression across an entire organ. Adeno-associated virus (AAV) has emerged as an extremely versatile vehicle for gene delivery due to its persistence and ability to transduce a variety of tissues [2-5]. Investigators have demonstrated successful intravenous (IV) AAV delivery via the superficial temporal vein in newborn mice or the jugular vein, portal vein and tail vein in adult mice [6-9]. Systemic IV delivery routes are particularly well suited when an extensive biodistribution of transgene expression is advantageous. In order to achieve thorough perfusion in one specific tissue but not throughout the entire body, greater control may be required.

In addition to the physical delivery route, another way to confine expression is by choosing a gene delivery vehicle with a high natural tropism for the tissue of interest. Several groups have demonstrated the unique ability of the AAV9 capsid to yield extraordinarily high levels of transgene expression in the heart [8-11]. Each of these groups using their respective delivery routes and detection systems also observed various levels of expression in other tissues. These data demonstrate that selection of a particular delivery vehicle alone is not enough to isolate transgene expression.

One approach to further increase specificity is to select a promoter that naturally drives expression of a particular gene in the tissue/s of interest. The objective of the current study was to identify which one of a group of promoters confers the greatest degree of cardiac specific expression and concurrently, which provides a broad biodistribution of expression across both cardiac and skeletal muscle.

**Figure 1:**
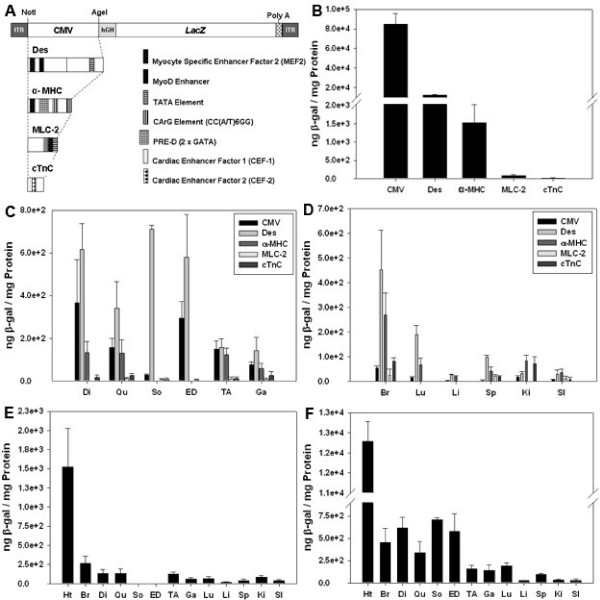
A) Construct Diagrams. Promoters were switched into the backbone by replacement of the CMV promoter between the first NotI and AgeI sites. The Des construct was created using primers against human genomic DNA (forward [F] Des enhancer primer containing NotI) ATA AGA ATG CGG CCG CAC CCA TGC CTC CTC AGG TA, (reverse [R] Des enhancer primer containing XhoI) CCG CTC GAG GGT GGG GCC TCA AGT TTA T, ([F] Des promoter primer containing XhoI) CCG CTC GAG ATA ACC AGG GCT GAA AGA, ([R] Des promoter primer containing AgeI) TGTA CCG GTG ACG GCG CGG GCG AGG CT. The α-MHC construct was created by amplifying human genomic DNA: ([F] containing NotI) ATA AGA ATG CGG CCG CCC AGT TGT TCA ACT CAC CCT TCA and ([R] containing AgeI) TGT ACC GGT GGG TTG GAG AAA TCT CTG ACA GCT. The MLC-2 construct was created by replacing the backbone with the previously described rat MLC-2 promoter[20]. ([F] containing NotI) ATA AGA ATG CGG CCG CGA CCC AGA GCA CAG AGC ATC GT ([R] containing AgeI) TGT ACC GGT GAA TTC AAG GAG CCT GCT. The cTnC construct was created by amplifying human genomic DNA: ([F] containing Not1) ATA AGA ATG CGG CCG CCA GCC TGA GAT CAC TGG GAC CAG A ([R] containing Age1) TGT ACC GGT CCA TGC TGG CGG CTC ACA GGA. 5 × 10^10^vg/mouse was administered (n = 6 per promoter group) [23]. Tissue lysates were assayed using the Galacto-Star chemiluminescence reporter gene assay system (Tropix, Inc., Bedford, MA, USA). Protein concentrations were determined using the Bio-Rad DC protein assay kit (Bio-Rad, Hercules, CA, USA). B) β-galactosidase (β-gal) expression levels show that CMV provides the greatest amount of expression in the heart followed by Des and α-MHC. C) β-gal levels in skeletal muscle including the diaphragm were highest in mice that were administered the Des construct. (Di, diaphragm; Qu, quadriceps; So, soleus; ED, extensor digitorum longus; TA, tibialis anterior; Ga, gastrocnemius) D) Evaluation by β-gal assay of non-heart, non-skeletal muscle tissues revealed highest expression levels in brain and lung from mice injected with the Des construct. (Ht, heart; Br, brain; Lu, lung; Li, liver; Sp, spleen; Ki, kidney; SI, small intestine) E) and F) β-gal levels and biodistribution profiles from α-MHC and Des construct injected mice (respectively).

Five promoters were compared: cytomegalovirus immediate-early gene promoter (CMV), human desmin (Des), human alpha-myosin heavy chain (α-MHC), rat myosin light chain 2 (MLC-2) and human cardiac troponin C (cTnC) (Figure 1A). Each promoter-*LacZ *construct was flanked by the inverted terminal repeats of AAV2 and packaged into the AAV9 capsid to yield rAAV2/9-promoter-*LacZ*. The constructs were tested by transfection and subsequently infection of both differentiated and undifferentiated C2C12 cells and a previously described immortalized cardiomyocyte line [12] to confirm promoter function (data not shown).

The CMV construct (690 base pairs [bp]) used in these experiments was from Stratagene^® ^(rAAV2/9-CMV-*LacZ*). It contains 5 cyclic AMP response-element binding protein (CRE-BP – a member of the leucine zipper family of transcription factors) binding sites as well as 4 NFkappaB binding sites. The CMV promoter confers virtually ubiquitous expression throughout the body except in the liver where it becomes inactive once the initial inflammatory phase passes [13]. The goal of this study was to identify alternatives to the CMV promoter.

The human desmin construct (354 bp) described in this study (rAAV2/9-Des-*LacZ*) contains both a myocyte specific enhancer factor 2 (MEF2) and a MyoD enhancer element. A TATA box was also added to increase transcription specificity. The primers were designed using the Catalogue of Regulatory Elements [14]. This promoter normally drives expression of desmin, a major intermediate filament protein essential for maintaining the functional and structural integrity of muscle [15]. Analysis of human tissues has revealed desmin expression in cerebellum, endometrium, skeletal muscle, neuronal cells of the lateral ventricle and heart [16]. Desmin may be a useful promoter to incorporate when performing systemic transgene delivery in myopathies. Diverse targets could include cardio skeletal muscle and even neurons.

The human α-MHC promoter (363 bp) (rAAV2/9-α-MHC-*LacZ*) construct contains a MEF2 region, a PRE-D sequence (tandem GATA sites separated by 4 bp), and 2 CArG elements. The primers were designed using the Catalogue of Regulatory Elements [14]. Myosin heavy chain is the most abundant component of the cardiac sarcomere [17]. We therefore hypothesized that it would be a highly specific cardiac promoter. The α-MHC protein is a "fast" ATPase myosin and is located in the thick filaments of myofibrils. It is important for cardiomyocyte contraction and relaxation [18]. In mice, the α-MHC protein is expressed in cardiac atrium and ventricles as well as skeletal muscle. In humans, α-myosin expression is restricted to the atria, and the β-MHC isoform ("slow" ATPase myosin) is the most predominant in ventricles [17]. Previous *in vitro *experiments with rat neonatal cardiomyocytes and mouse studies have shown that the MHC promoter preferentially expresses in cardiac tissue more than skeletal muscle [19].

The promoter incorporated into the rat MLC construct described here, (479 bp) (rAAV2/9-MLC-*LacZ*) has been previously described by Henderson *et. al. *and was originally designed from cardiac MLC-2 [20]. While various isoforms exist, the cardiac MLCs can be divided into 2 types: MLC-1, non-phosphorylatable and MLC-2, phosphorylatable. Ca^2+ ^dependent phosphorylation of MLC-2 by MLC kinase provides an important a regulatory function in the contraction of cardiac, skeletal and smooth muscle [20]. Two different isoforms of MLC-2 are typically expressed in the atria and ventricles of the mammalian heart during embryonic development and maturation, and later in the adult heart [21]. The rat-derived MLC promoter characterized here contains a CArG box as well as a MyoD enhancer sequence.

The human cTnC construct (rAAV2/9-cTnC-*LacZ*) (175 bp) contains 2 cardiac enhancer factor (CEF) sites. The primers for this promoter were designed using the Catalogue of Regulatory Elements [14]. The CEF sites of the human cTnC bind cardiac specific nuclear proteins [22]. CEF-1 specifically binds the GATA-4 protein in addition to a currently uncharacterized nuclear protein complex that is also bound by CEF-2. The cardiac troponin C (cTnC) gene produces identical transcriptsin both slow twitch skeletal muscle as well as the heart. It binds Ca^2+ ^and prevents actin-myosin interaction in resting muscle. We hypothesized that elements from the troponin promoter, which is found in both heart and skeletal muscle, would drive expression in all striated muscle.

BALB/c neonatal mice received 5 × 10^10 ^vector genomes (vg) of each vector via single injections to the superficial temporal vein (n = 6 per promoter group). After 4 weeks the tissues were harvested and expression levels were evaluated by β-galactosidase enzyme assay. The highest levels of expression were observed in hearts of animals injected with the CMV, Des or α-MHC constructs (Figure 1B). Expression levels in hearts of animals injected with viruses containing either the MLC-2 or cTnC promoters were comparatively weak.

Analysis of expression in skeletal muscles including the diaphragm (Figure 1C) revealed the strongest β-galactosidase expression levels were from the Desmin promoter. The CMV promoter showed the next highest expression levels followed by the other promoters. Analysis of non-heart, non-skeletal muscle tissues (Figure 1D) showed that the Des promoter produced the highest expression levels in the brain.

Comparing biodistribution profiles of each promoter individually showed that of those included in this study, the α-MHC is the most cardiac specific (Figure 1E) and the Des construct is well suited for achieving transgene expression in both heart and skeletal muscle (Figure 1F). Importantly, the Des promoter also showed expression levels in the brain that were similar to those found in muscle. This is a key attribute when gene therapy is applied to Pompe Disease where a neuronal as well as a muscular component has been observed [7].

The biodistribution of viral vector genomes from mice injected with the Des construct was assessed by real time PCR on DNA isolated from each tissue. This measurement is indicative of viral genome location and is independent of the specific promoter being delivered. The vector genome biodistribution profile was very similar to that previously described for AAV9 [8] confirming that the variance in expression profiles result from the different promoters being employed. Heart contained the highest concentration at 19 ± 2 copies per cell followed by the diaphragm with approximately 0.13 ± 0.007 copies per cell. All other tissues contained less than 0.01 copies per cell.

Ultimately, these data serve as a characterization of 5 different promoters and their respective behavior *in vivo *following AAV9 mediated gene delivery. Our data indicate that the α-MHC promoter confers the most cardiac specific expression and that the Des promoter provides expression in a variety of tissues. The combined use of application tailored promoters and delivery vehicles with well-understood tropisms augment the control investigators have over expression of their transgene of interest and thereby increase the over-all safety of therapeutic gene delivery.

## Competing interests

The Johns Hopkins University, the University of Florida, B.J.B., C.A.P., and C.S.M. could be entitled to patent royalties for inventions described in this article.

## Authors' contributions

CAP participated in the design of the study, performed the injections, ran the β-galactosidase enzyme detection assays and drafted the manuscript. YS designed and cloned the plasmids and harvested tissues. BDT performed the RNA transcript analysis and helped to draft the manuscript. CSM participated in the design of the study and helped to draft the manuscript. BJB participated in the design of the study and reviewed the manuscript. All authors read and approved the final manuscript.
