# Tissue specific promoters improve specificity of AAV9 mediated transgene expression following intra-vascular gene delivery in neonatal mice

**DOI:** 10.1186/1479-0556-7-3

**Published:** 2009-02-04

**Authors:** Christina A Pacak, Yoshihisa Sakai, Bijoy D Thattaliyath, Cathryn S Mah, Barry J Byrne

**Affiliations:** 1 Powell Gene Therapy Center, College of Medicine, University of Florida, 1600 SW Archer Road, Gainesville, FL 32610-0266, USA

## Correction

Since publication of our article [1], it has come to our attention that the alpha-myosin heavy chain promoter was incorrectly identified as being of human origin. The correct origin of the promoter was from mouse.

The corrections within the text are as follows:

Under the heading **Findings**, 5 paragraph, line 3: "human alpha-myosin heavy chain" should be "murine alpha-myosin heavy chain."

Under the heading **Findings**, 8^th ^paragraph, line 1: "The human a-MHC promoter" should be "The murine a-MHC promoter"

Figure 1, Figure legend, line 6; "...created by amplifying human genomic DNA" should be "... created by amplifying mouse genomic DNA."

The authors regret the error and any inconveniences to the readers. The results and conclusions of this article remain unchanged.
